# Measurement of serum total and free prostate-specific antigen in women with colorectal carcinoma

**DOI:** 10.1038/sj.bjc.6600049

**Published:** 2002-01-21

**Authors:** N Duraker, D Can, M Parıltı

**Affiliations:** Fifth Department of Surgery, SSK Okmeydanı Training Hospital, PO Box 80434, İstanbul, Turkey; Third Department of Surgery, SSK Okmeydanı Training Hospital, PO Box 80434, İstanbul, Turkey; Department of Nuclear Medicine, SSK Okmeydanı Training Hospital, PO Box 80434, İstanbul, Turkey

**Keywords:** PSA, free PSA, woman, female serum, colorectal cancer

## Abstract

We investigated the diagnostic value and the relationship with clinicopathological features of total and free prostate-specific antigen by measuring the concentrations of these markers in the sera of 75 women with colorectal carcinoma and in 30 healthy women. Measurements were performed by immunoradiometric assay which utilizes monoclonal and polyclonal anti-prostate-specific antigen antibodies; the lowest detection level for both markers was 0.01 ng ml^−1^. Free prostate-specific antigen levels were significantly higher in women with colorectal carcinoma than healthy women (*P*=0.006). The percentage of free prostate-specific antigen predominant (free prostate-specific antigen/total prostate-specific antigen >50%) subjects was 20% in colorectal carcinoma patients and 3.3% in healthy women (*P*=0.035). Cut-off values were 0.34 ng ml^−1^ for total prostate-specific antigen and 0.01 ng ml^−1^ for free prostate-specific antigen. In women with colorectal carcinoma, total prostate-specific antigen positivity was 20% and free prostate-specific antigen positivity was 34.6%. When compared to negatives, total prostate-specific antigen positive patients had a lower percentage of well-differentiated (*P*=0.056) and early stage (stages I and II) tumours (*P*=0.070). However, patients with predominant free prostate-specific antigen, had a higher percentage of well-differentiated (*P*=0.014) and early stage tumours (*P*=0.090) than patients with predominant bound prostate-specific antigen. In conclusion, although the sensitivity of free prostate-specific antigen predominancy is low (20%), in distinguishing women with colorectal carcinoma than healthy women, its specificity is high (96.7%). Free prostate-specific antigen predominancy tends to be present in less aggressive tumours. These findings may indicate clinical significance of preoperative measurement of serum total and free prostate-specific antigen in women with colorectal carcinoma.

*British Journal of Cancer* (2002) **86**, 203–206. DOI: 10.1038/sj/bjc/6600049 www.bjcancer.com

© 2002 The Cancer Research Campaign

## 

It has been shown by immunofluorometric and immunohistochemical assays that prostate-specific antigen (PSA) which is widely utilized for the diagnosis and management of prostate cancer, is not specific for the prostate gland itself but it also exists in breast, ovarian, endometrial, kidney, adrenal and salivary gland cancer tissues (Diamandis and Yu, 1997). Recently, molecular forms of PSA (α_1_-antichymotripsin-bound PSA and free PSA) have been investigated in the sera of women with benign and malignant breast diseases and it has been reported that existence of free PSA as the major molecular form may be of diagnostic importance in female breast cancer (Borchert *et al*, 1997; Black *et al*, 2000).

The production of PSA in the prostate is up-regulated by androgens, through the androgen receptor (Young *et al*, 1991; Henttu *et al*, 1992; Luke and Coffey, 1994). The gene expression and protein production of PSA in nonprostatic tissues are under the regulation of steroid hormones via their receptors; androgens, glucocorticoids, mineralocorticoids and progestins up-regulate the PSA production; oestrogens down-regulate indirectly the PSA production induced by androgens (Yu *et al*, 1994b; Zarghami *et al*, 1997). The existence of androgen, progesterone and oestrogen receptors has been demonstrated in colorectal cancer tissues (Meggouh *et al*, 1991; Singh *et al*, 1993; Korenaga *et al*, 1997, 1998). The relation between colorectal cancer and steroid hormone receptors may indicate the existence of PSA in cancer tissues.

It has been reported that PSA expression is not found (van Krieken, 1993) or whenever existed, it is found in trace amounts (Levesque *et al*, 1995) in colon cancer tissues. In a case report (Yamamoto *et al*, 1997), in a patient with colon adenocarcinoma who showed no evidence of prostate cancer, there were high PSA levels preoperatively that returned to normal level after total removal of the cancer.

In this study, to our knowledge for the first time in the literature, preoperative serum total and free PSA concentrations were measured in women with colorectal carcinoma, and the relationship with clinicopathological features and the diagnostic value of these markers were investigated.

## MATERIALS AND METHODS

### Patients

Between June 1998 and December 2000, serum total and free PSA levels were measured preoperatively in 75 women with colorectal carcinoma and with no other pathology (mean age 60 years, range 27–82 years), who were operated at SSK Okmeydanı Training Hospital, İstanbul, and in 30 ‘healthy’ women (mean age 40.4 years, range 17–69 years). All women included in the study had been informed and their approvals were obtained. In 69 patients, the tumour could be resected while six tumours were unresectable due to adjacent organ and structure invasion. The location, size and histologic features of the tumour were reported by the referred pathologist. The size and histologic features of the unresected tumours were not included in the evaluation. Venous invasion was not examined in one of the resected tumours. Histologic type was divided in two subgroups as well-differentiated adenocarcinoma (15 cases) and the others. In the second group, there were 47 moderately and three poorly differentiated adenocarcinomas, three mucinous carcinomas and one signet ring cell carcinoma. The depth of invasion and staging of the disease were assessed according to TNM system (Fleming *et al*, 1997). Two patients with unresected tumours and with no distant metastasis were included in advanced stage disease (stages III and IV).

### PSA assays

Peripheral venous blood samples were drawn and centrifugated in healthy women and 2 to 24 h before the operation in the colorectal cancer patients. The collected sera were kept in −20°C until analyzed. Total PSA measurements were performed with Coat-A-Count® PSA IRMA (DPC, Los Angeles, CA, USA) kit and free PSA measurements were performed with Coat-A-Count® Free PSA IRMA (EURO/DPC Ltd, UK) kit, by utilizing solid-phase sequential immunoradiometric assay which is based on monoclonal and polyclonal anti-PSA antibodies. Lower detection limit for each tumour marker was calculated as 0.01 ng ml^−1^ from the calibration curve by interpolation.

### Statistics

The serum total and free PSA levels of 30 healthy women were arranged in sequence from the lowest to highest and the marker concentrations of the 28th woman were accepted as cut-off values (specificity 93.3%). Marker levels exceeding these cut-off values were considered as positive. Cases with a free PSA/total PSA ratio over 50% were titled ‘free PSA predominant subject’.

Mann–Whitney *U*-Wilcoxon Rank Sum W test was used for comparisons of total PSA or free PSA levels between colorectal carcinoma patients and healthy women. The effect of age on total PSA and free PSA levels was investigated using Spearman correlation analysis. Fisher exact test was used for comparison of free PSA predominancy between carcinoma patients and healthy women. Chi-square test and Fisher exact test were used to assess the association between clinicopathological factors and total PSA positivity, free PSA positivity or free PSA predominancy. *P*-values of less than 0.05 were considered to be statistically significant.

## RESULTS

### PSA levels

As shown in Table 1

**Table 1:**
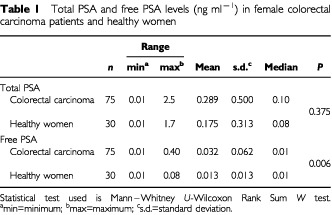
Total PSA and free PSA levels (ng ml^−1^) in female colorectal carcinoma patients and healthy women

, free PSA levels in women with colorectal carcinoma were significantly higher than healthy women (*P*=0.006) whereas no significant association existed in total PSA levels of both groups. The ratio of free PSA predominant subjects were 20% (15 out of 75) in colorectal carcinoma group and 3.3% (one out of 30) in the healthy group (*P*=0.035). In colorectal carcinoma patients and healthy women, total PSA and free PSA levels decreased as age increased, though the correlations were not significant in Spearman analysis (data not shown).

### Association between clinicopathological factors and PSA positivities

The cut-off values for total PSA and free PSA were 0.34 ng ml^−1^ and 0.01 ng ml^−1^ respectively. Total PSA positivity was 20% (15 out of 75) and free PSA positivity was 34.6% (26 out of 75) in women with colorectal carcinoma. As shown in Table 2

**Table 2:**
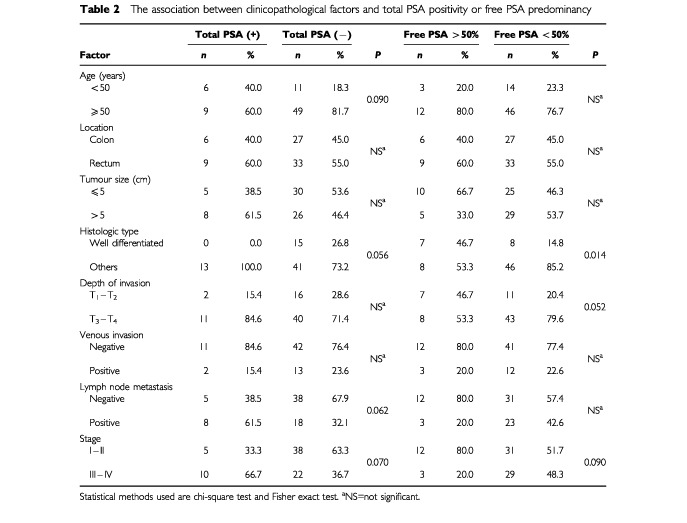
The association between clinicopathological factors and total PSA positivity or free PSA predominancy

, there was no significant association between total PSA positivity and location and size of the tumour, depth of invasion or venous invasion. The ratio of women younger than 50 years was slightly higher in total PSA positives than negatives, but the difference was not significant (*P*=0.090). In total PSA positive patients, no well-differentiated tumour existed, whereas 26.8% of total PSA negatives had well-differentiated tumours (*P*=0.056). Compared to negatives, in total PSA positive group, the ratio of the patients with lymph node metastasis and advanced stage disease had a tendency to increase (*P*=0.062 and *P*=0.070 respectively). There was no significant association between clinicopathological variables and free PSA positivity (data not shown).

Table 2 shows that no significant association existed between free PSA predominancy and age, location and size of the tumour, venous invasion or lymph node involvement. In free PSA predominant patients, compared to patients with α_1_-antichymotripsin bound PSA as the predominant molecular form, the ratio of well- differentiated tumours were significantly higher (*P*=0.014); the ratios of T_1_–T_2_ tumours (*P*=0.052) and early stage disease (stages I and II) (*P*=0.090) were slightly higher.

## DISCUSSION

In this initial report assessing serum total and free PSA levels in women with colorectal carcinoma, total PSA levels were found to be higher than healthy women but the difference was not significant. On the other hand free PSA levels were significantly higher in women with colorectal carcinoma.

PSA measurements in nonprostatic carcinomas have been conducted mostly in women with breast cancer. It has been shown that serum total PSA levels in women with breast cancer were significantly higher than healthy women, and in women with breast cysts were significantly higher than women with breast cancer and serum free PSA levels in breast cancer patients were significantly higher than healthy women (Black *et al*, 2000). Another study revealed serum total PSA concentrations to be significantly lower in women with breast cancer than women with benign breast disease and no significant difference was found between cancer patients and normal women; free PSA levels showed no difference in the groups (Borchert *et al,* 1997). In another study, no significant difference in serum total PSA levels was found between breast cancer patients and women with benign breast disease or healthy women (Romppanen *et al*, 1999).

Total PSA is the sum of different molecular forms of PSA existing in serum, that is, free (non-complexed) PSA and PSA complexed to α_1_-1-antichymotripsin. Recently, it has been demonstrated that free PSA/total PSA ratio in prostate cancer patients is lower than patients with benign prostatic hyperplasia and the percentage of free PSA improves specificity and sensitivity of prostate cancer diagnosis (Catalona *et al*, 1995; Törnblom *et al*, 1999; Veltri and Miller, 1999). Cut-off values proposed for per cent free PSA have ranged from 17 to 25% (Kamoi and Babaian, 1999).

Studies conducted in women with breast cancer revealed contrary results. It was demonstrated that 44% of women with breast cancer and 58% of women with benign breast disease had serum free PSA as the major molecular form, whereas normal women had PSA bound to α_1_-antichymotripsin as the major molecular form, and it was suggested that the ratio of free PSA/bound PSA might have value for diagnosis of breast diseases including breast cancer (Borchert *et al*, 1997). In another study (Black *et al*, 2000), it was demonstrated that the percentage (20%) of breast cancer patients with free PSA as the predominant molecular form (>50% of total PSA) in serum was significantly higher than that of healthy women (3%) or women with benign breast disease (4%), and it was stated that although free PSA as the predominant molecular form has high specificity (96%), its clinical utility is limited due to low sensitivity (20%).

We concluded similar results as with the study mentioned above (Black *et al*, 2000), in our study. The percentage of free PSA predominant subjects (free PSA/total PSA >50%) in women with colorectal carcinoma was 20%, which was significantly higher than healthy women (3.3%). Although the sensitivity of free PSA predominancy was low (20%) in distinguishing women with colorectal carcinoma than healthy women, the specificity was higher (96.7%) which justifies further investigations to clarify its clinical significance.

In our study, although serum total and free PSA levels were decreased as age increased both in women with colorectal carcinoma and healthy women, the correlations were not significant; the percentage of women older than 50 years was slightly lower in total PSA positive patients than negatives. In one study serum total PSA levels were found to decrease significantly with ageing in both healthy women and women with breast cancer (Romppanen *et al*, 1999). However, in another study no significant correlation was found between serum total PSA levels and age in normal women while a negative correlation was demonstrated in women with breast cancer (Borchert *et al*, 1997). As in the explanation of the decrease of PSA expression with ageing in breast cancer tissue (Yu *et al*, 1994a), the decrease in PSA production with ageing might be due to the decrease of ovarian hormones which mediate PSA production by binding to the steroid receptors found in colorectal cancer tissue.

The cut-off values providing the specificity rate of 93.3% in our series, were 0.34 ng ml^−1^ for total PSA and 0.01 ng ml^−1^ for free PSA. The cut-off value in breast cancer diagnosis for total PSA is 30 ng ml^−1^ (Borchert *et al*, 1997; Romppanen *et al*, 1999) which is 10 times lower than the value we found. This discrimination may be due to the difference in the sensitivities of PSA assays. With our cut-off values, in our series, total PSA positivity was 20% and free PSA positivity was 34.6% in women with colorectal carcinoma. With a cut-off value of 30 ng ml^−1^ total PSA positivity rates were reported to be 6.5% (Borchert *et al*, 1997) and 5.6% (Romppanen *et al*, 1999) in women with breast cancer.

We did not find any significant association with serum total PSA levels and the location, tumour size, depth of wall invasion or venous invasion. There was no well-differentiated tumour in total PSA positive patients while 26.8% of the negative patients had well-differentiated tumours, the difference was nearly significant. In total PSA positive patients compared to negatives, there was a tendency to be in advanced stage disease and lymph node metastasis. No significant association could be found between free PSA positivity and the clinicopathological features. Well-differentiated tumours were significantly higher in patients with predominant free PSA; there was a tendency to be T_1_–T_2_ and early stage tumours in this group. There was no significant association with free PSA predominancy and other clinicopathological factors.

No significant relationship was found in breast cancer patients between serum total PSA levels and histologic grade or disease stage (Borchert *et al*, 1997; Romppanen *et al*, 1999). In another study no association was found between histologic grade and serum total PSA or free PSA predominancy (Black *et al*, 2000). In accordance with our results, it has been shown that in prostate cancer patients, the aggressiveness of the histologic grade increased significantly as the percentage of free PSA/total PSA decreased (Li *et al*, 1999).

In summary, with a specificity rate of 93.3%, the sensitivity rate for serum total PSA was 20% and for serum free PSA, it was 34.6% in women with colorectal carcinoma. Free PSA predominancy (free PSA/total PSA >50%) showed a sensitivity rate of 20% and a specificity rate of 96.7% in distinguishing women with colorectal carcinoma than healthy women. Although total PSA positivity had a tendency to be present in aggressive histologic types and in advanced stage tumours, free PSA predominancy had a tendency to be present in well-differentiated and early stage tumours.

In conclusion, this data reveals that preoperative serum total and free PSA measurements may offer clinical significance in women with colorectal carcinoma and there is need for new studies including wider patient and control groups, utilizing ultrasensitive PSA assays whose lowest detection limit is lower.
